# Statewide program to promote institutional delivery in Gujarat, India: who participates and the degree of financial subsidy provided by the Chiranjeevi Yojana program

**DOI:** 10.1186/s41043-016-0039-z

**Published:** 2016-01-27

**Authors:** Kristi Sidney, Veena Iyer, Kranti Vora, Dileep Mavalankar, Ayesha De Costa

**Affiliations:** 1 Public Health Sciences, Karolinska Institutet, Widerströmska, Tomtebodavägen 18A, plan 4, SE-171 77 Stockholm, Sweden; 2 Indian Institute of Public Health Gandhinagar, Public Health Foundation of India, Ahmedabad, Gujarat India

**Keywords:** Maternal health, Out-of-pocket expenditures, Demand-side financing, Chiranjeevi Yojana, Public-private partnership, India

## Abstract

**Background:**

The Chiranjeevi Yojana (CY) is a large public-private partnership program in Gujarat, India, under which the state pays private sector obstetricians to provide childbirth services to poor and tribal women. The CY was initiated statewide in 2007 because of the limited ability of the public health sector to provide emergency obstetric care and high out-of-pocket expenditures in the private sector (where most qualified obstetricians work), creating financial access barriers for poor women. Despite a million beneficiaries, there have been few reports studying CY, particularly the proportion of vulnerable women being covered, the expenditures they incur in connection with childbirth, and the level of subsidy provided to beneficiaries by the program.

**Methods:**

Cross-sectional facility based the survey of participants in three districts of Gujarat in 2012–2013. Women were interviewed to elicit sociodemographic characteristics, out-of-pocket expenditures, and CY program details. Descriptive statistics, chi square, and a multivariable logistic regression were performed.

**Results:**

Of the 901 women surveyed in 129 facilities, 150 (16 %) were CY beneficiaries; 336 and 415 delivered in government and private facilities, respectively. Only 36 (24 %) of the 150 CY beneficiaries received a completely cashless delivery. Median out-of-pocket for vaginal/cesarean delivery among CY beneficiaries was $7/$71. The median degree of subsidy for women in CY who delivered vaginally/cesarean was 85/71 % compared to out-of-pocket expenditure of $44/$208 for vaginal/cesarean delivery paid by non-program beneficiaries in the private health sector.

**Conclusions:**

CY beneficiaries experienced a substantially subsidized childbirth compared to women who delivered in non-accredited private facilities. However, despite the government’s efforts at increasing access to delivery services for poor women in the private sector, uptake was low and very few women experienced a cashless delivery. While the long-term focus remains on strengthening the public sector’s ability to provide emergency obstetric care, the CY program is a potential means by which the state can ensure its poor mothers have access to necessary care if uptake is increased.

**Electronic supplementary material:**

The online version of this article (doi:10.1186/s41043-016-0039-z) contains supplementary material, which is available to authorized users.

## Background

Despite the global maternal mortality ratio (MMR) declining from 380 maternal deaths per 100,000 live births in 1990 to 210 deaths in 2013 [1], maternal deaths still remain high in some countries such as India. Almost a fifth of the 287,000 annual maternal deaths occur in India [2–5].

It is known that skilled birth attendance and access to quality emergency obstetric care (EmOC) are critical to the reduction of maternal mortality [6, 7]. Institutional childbirth has been advocated and adopted by governments all over the world, including India, as a strategy to reduce maternal mortality. Considering the unpredictable occurrence of life-threatening obstetric complications, the assumption is that a facility birth will provide a woman access to skilled birth attendance and EmOC, facilitating the management of complications that could ultimately lead to a reduction in mortality [8].

Although governments in many low middle income countries actively encourage facility-based childbirth for this reason, the capacity of public health facilities to provide life-saving EmOC is limited because of structural weaknesses in the health system including a lack of qualified human resources and shortages of infrastructure and supplies [9]. Such a situation exists in the public health system in many parts of India and in the Western Indian state of Gujarat. The public health sector has an extreme shortage of qualified obstetricians [10] and hence little capacity to provide EmOC. However, in comparison, there are over 1500 qualified obstetricians [11] practicing in the for-profit private health sector. This sector operates largely on the basis of out-of-pocket (OOP) payments from users.

The relationship between poverty and maternal death is well known [12]. Recent studies in South Asia [13, 14] have highlighted OOP expenditures for poor women as a barrier to seeking childbirth services in a health facility. In 2005–2006, only 13 % of India’s poorest women gave birth in a health facility providing EmOC, while the corresponding figure for the wealthiest women was 84 % [15]. Poor/tribal women (who bear the brunt of maternal morbidity and mortality) face financial barriers to accessing functional EmOC services in the country as these services are largely concentrated in the for-profit private sector [16, 17]. This inequity emphasizes the importance of developing strategies that remove financial barriers to maternal delivery services and enable poor women to receive proper care where it is available.

In order to minimize financial barriers and provide poor/tribal women access to the available EmOC in the private sector, the Government of Gujarat initiated a voucher-like program, Chiranjeevi Yojana (CY, a scheme for long life). Under this public-private partnership, qualified private obstetricians are paid by the state government to provide a cashless delivery for poor/tribal women within the state [18].

Most voucher-like programs worldwide are small and managed by non-governmental organizations or donors [19]. CY in comparison is a large statewide voucher-like program run and financed entirely by the government. Despite nearly a million beneficiaries [20], there have been few reports critically studying the CY public-private partnership [21–27]. While a small pilot evaluation was performed in 2006 [21], only three studies were implemented since the program was rolled out statewide. Two studies examined the impact of CY on increasing institutional delivery [23, 24], and the third was a qualitative study focusing on the perception and experience of private providers with regard to the CY program [27].

This paper aims to advance the state of knowledge on the CY program particularly by establishing the degree of uptake and the level of financial subsidy obtained by beneficiaries by (i) studying the proportion of eligible women who become CY beneficiaries and (ii) ascertaining OOP expenditures and the extent the CY program subsidized childbirth. This is relevant not only for researchers, implementers, and policy makers in India but also for other low-income settings where similar programs are being planned and implemented.

## Methods

### Study setting

Gujarat, India, has a population of 60.3 million [4], a per capita income 25 % higher than the national average, a MMR of 122 per 100,000 live births [2], and an infant mortality rate of 41 per 1000 live births [28]. The state is divided into 26 administrative districts, each with a population of 1–3 million [4]. It is considered one of the high-performing states in India with strong socioeconomic growth over the last decade and a 24 % reduction in MMR between 2004 and 2012 [2]. Sixty percent of all births in the state take place in the private sector [24].

#### The Chiranjeevi Yojana program

CY is a performance-based financing program that functions in the context of an existing strong private obstetric care sector in Gujarat, India. The rationale for the program has been described above. The state government pays accredited private facilities, run by qualified obstetricians, to provide free childbirth care to women from below poverty line (BPL) households and tribal women. BPL or tribal eligibility is identified by official documentation provided by a government authority [29]. All willing private obstetricians who met the basic requirements outlined by the government could apply to participate in the CY program.

The remuneration package at the time of the study was $5600 per 100 deliveries (described in Additional file 1). The package has been revised upwards periodically since the program’s inception. The payment structure creates an embedded disincentive for unnecessary cesareans as the provider receives a fixed payment per 100 deliveries regardless of the delivery mode. The program was implemented statewide in 2007 and has benefited almost a million women [20].

### Study design: a cross-sectional study performed in health facilities

#### Study area

Three districts, Sabarkantha, Dahod, and Surendranagar, were purposefully selected from diverse geographic areas. These districts had varying human development indices and different population compositions, i.e., varying proportions of tribes and populations living below the poverty line. As seen in Table 1, the eligible population for the program differed widely among the study districts, as did the number of accredited facilities.

**Table 1:** Characteristics of the study districts in Gujarat, India

District	Population (millions) [4]	Private facilities [37]^a^	CY facilities [3]	Eligible population (%) [42]	Crude birth rate [3]	Literacy rate (%) [4]
Sabarkantha	2.4	53	49	43	21	77
Dahod	2.1	13	14	88	24	61
Surendranagar	1.8	19	18	45	24	73

^a^Private facilities that provided comprehensive emergency obstetric care including cesarean sections and blood transfusions

### Data collection

#### Identifying facilities providing intrapartum care

An initial list of all public and private health facilities that routinely provided intrapartum care was obtained from the district public health officials. These facilities and local pharmacies were approached to identify any remaining private facilities that were not on the initial listing. The number of deliveries performed in the previous 3 months for each of the identified facilities was ascertained. Facilities that performed more than 30 deliveries in the previous 3 months were included in the study.

#### Study participants

Trained research assistants visited each of the study facilities for a consecutive 5-day period and interviewed women who gave birth at these facilities. A questionnaire was administered to the mother or a family member present in the facility before discharge. Basic sociodemographic characteristics, pregnancy and delivery details, OOP expenses, and whether they received the CY benefit were elicited. More specific details related to the delivery and complications experienced (when applicable) were obtained from a nurse on the labor ward. On average, the administration of the questionnaire took 25 min. During this period, research assistants also enquired whether the facility routinely performed cesarean sections and blood transfusions in the last 3 months or only vaginal deliveries. The study was performed between June 2012 and April 2013.

During the recruitment period, 1632 mothers delivered in the study facilities. Women were excluded from this study for the following reasons: (i) not eligible for the CY program (*n* = 409, 25 %), (ii) discharged from the facility before being recruited (*n* = 221, 14 %), or (iii) resided outside the province of Gujarat (*n* = 101, 6 %).

### Definitions

#### Eligibility criteria for women to be beneficiaries of the CY program

Women were considered eligible for the program if they reported possessing a government-issued BPL card, tribal certificate, or other officially accepted documentation as formal proof of poverty status.

#### Beneficiary status by place of delivery

Beneficiary status by place of delivery are grouped as follows: *CY beneficiary* (CYB): women who delivered in a facility participating in the CY program and reported receiving the CY benefit. *CY non-beneficiary* (CYNB): eligible women who delivered in a CY facility but did not receive the benefit. *Private non-beneficiary* (PNB): Eligible women who delivered in a non-accredited private facility and did not receive the CY benefit. *Government non-beneficiary* (GNB): eligible women who delivered in a government-run (public sector) facility and hence did not receive the CY benefit.

Facilities were classified into three groups depending on if they provided cesarean sections (CS) and blood transfusions (BT) in the previous last 3 months: *non-CS facility*: facilities that did not provide CS and only conducted vaginal deliveries. *CS facilities*: facilities that conducted both vaginal and CS deliveries but did not provide BT. *CS & BT facilities*: facilities that conducted vaginal and CS deliveries and provided BT.

### Background variables

1. Education: Women were categorized as having no formal education (i.e., never went to school) or having some formal education.
2. Caste or tribe: Women were divided into three groups, i.e., tribal (indigenous people), backward caste, and general (not backward caste). Backward castes are specially identified groups in the Indian constitution who have faced social discrimination historically and are still vulnerable. The constitution identifies these groups as they are recipients of positive affirmative action under the law [30]. Backward caste includes scheduled caste and other backward castes.
3. Household wealth: To assess household wealth, 20 household items, structural type of dwelling, and sanitation arrangements were included as used in the National Family Health Survey [15]. Principal component analysis was used to calculate a wealth index score, and then women were categorized into five wealth quintiles.
4. Direct obstetric complications: Intrapartum care complications were recorded from a staff member on the labor ward. Hemorrhage (antepartum, intrapartum, or postpartum) prolonged/obstructed labor, postpartum sepsis, and severe pre-eclampsia/eclampsia were all classified as complications.
5. CY program awareness was recorded as “yes” if the women reported knowledge of the CY program prior to delivery.

To study the OOP expenditures and the degree of subsidy provided by the program, we grouped expenses incurred by each mother as follows:

1. *Health facility expenditure:* Both direct and indirect medical expenditures incurred for childbirth in the facility were collected. Direct medical OOP expenditures included expenses for delivery, medicines, supplies, BT, laboratory investigations, and anesthesia. Indirect medical OOP expenses included admission fee, accommodation charge, and food. All health facility expenditures (direct and indirect) are theoretically covered by payments to the obstetrician under the CY program, so that a CY beneficiary receives cashless service for their delivery.
2. *Informal payments* were expenditures reported as ‘rewards’ paid by the women/families to the staff for assisting their care.
3. *Transportation costs* included all costs associated with reaching the health facility for delivery.

#### Degree of subsidy provided by the CY program

The assumption was made that the expense paid for delivery by PNB was the current market price for childbirth services in the private sector. In the absence of the CY program, this would be the minimum price that a mother would have paid OOP if she delivered in the private sector. We calculated the extent to which each mother was subsidized by participating in the CY program shown below.

$$ \mathrm{Subsidy}\;\%\ \mathrm{f}\mathrm{o}\mathrm{r}\ \mathrm{vaginal}\ \mathrm{delivery}:\left[1-\left(\frac{\mathrm{Median}\ \mathrm{C}\mathrm{Y}\mathrm{B}\ \mathrm{Health}\ \mathrm{f}\mathrm{acility}\ \mathrm{expenditure}\ \mathrm{f}\mathrm{o}\mathrm{r}\ \mathrm{vaginal}\ \mathrm{delivery}+\mathrm{transportation}\ \mathrm{cost}\ }{\mathrm{Median}\ \mathrm{P}\mathrm{N}\mathrm{B}\ \mathrm{Health}\ \mathrm{f}\mathrm{acility}\ \mathrm{expenditure}\ \mathrm{f}\mathrm{o}\mathrm{r}\ \mathrm{vaginal}\ \mathrm{delivery}+\mathrm{transportation}\ \mathrm{cost}}\right)\right]\times 100\% $$

$$ \mathrm{Subsidy}\;\%\ \mathrm{f}\mathrm{o}\mathrm{r}\ \mathrm{C}\mathrm{S}\ \mathrm{delivery}:\left[1-\left(\frac{\mathrm{Median}\ \mathrm{C}\mathrm{Y}\mathrm{B}\ \mathrm{Health}\ \mathrm{f}\mathrm{acility}\ \mathrm{expenditure}\ \mathrm{f}\mathrm{o}\mathrm{r}\ \mathrm{C}\mathrm{S}\ \mathrm{delivery}+\mathrm{transportation}\ \mathrm{cost}\ }{\mathrm{Median}\ \mathrm{P}\mathrm{N}\mathrm{B}\ \mathrm{Health}\ \mathrm{f}\mathrm{acility}\ \mathrm{expenditure}\ \mathrm{f}\mathrm{o}\mathrm{r}\ \mathrm{C}\mathrm{S}\ \mathrm{delivery}+\mathrm{transportation}\ \mathrm{cost}}\right)\right]\times 100\% $$

#### Analysis

Descriptive statistics were used to describe the study sample by place of delivery. Chi square was used to identify significant differences between characteristics of women who delivered in an accredited CY facility and the other two groups (PNB and GNB). Simple proportions were used to describe the proportion of eligible women who became CYB and CYNB in an accredited CY facility. A multivariable logistic regression was performed to identify predictors of receiving the CY benefit within an accredited CY facility. The median and interquartile range (IQR) for health facility expenditures was stratified by vaginal and CS deliveries. Since the health expenditures were not normally distributed, the non-parametric Wilcoxon signed rank test was used to detect differences between different groups of women. Informal payments and transportation costs were also described. The percentage subsidy provided was calculated for each individual CYB and expressed as a median for the cohort.

The study was described to all study participants. Written informed consent was obtained from the participants before they were enrolled in the study and responded to the questionnaire. Ethical approval was granted by the Indian Institute of Public Health, Gandhinagar, Gujarat, India, with the ethical approval number TRC-IEC No:23/2012 and Karolinska Institutet:2010/1671–31/5.I.

## Results

One hundred fifty-eight public and private facilities were identified in the initial listing process. Among those facilities, 21 did not perform a delivery during the 5-day recruitment period and eight declined to participate in the study. As depicted in Table 2, the study participants delivered in 129 different facilities within the three study districts; 37 accredited CY private, 36 government, and 56 non-CY-accredited private facilities. Among the 129 facilities, 48 (37 %) did not perform CS, 8 (6 %) conducted only CS but not BT, and 73 (57 %) performed both CS and BT in the last 3 months. The majority (86 %, 31/36) of government facilities did not provide CS or BT while most private facilities (73 %, 68/93) provided both services.

**Table 2:** Access to emergency interventions (cesarean sections and blood transfusions) performed by facility type (*n*=129). Column % presented

	Accredited CY private facility	Non-accredited CY private facility	Government facility
Non-CS facility	5 (14)	12 (21)	31 (86)
CS facility	3 (8)	5 (9)	0 (0)
CS & BT facility	29 (78)	39 (70)	5 (14)

*CS* cesarean sections, *BT* blood transfusions

**Fig. 1:**
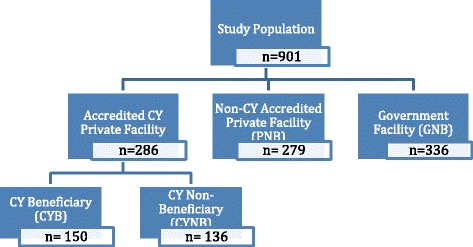
Study sample by place of delivery and receiving the CY benefit

As shown in Fig. 1, the final study sample included 901 women who met the CY program eligibility criteria of being BPL or tribal. Of these eligible women, 286 delivered in a facility that participated in the CY program, 150 (16 %) were CYB, and 136 were CYNB. Of the remaining eligible non-beneficiaries, 336 delivered in a government facility (GNB) and 279 delivered in a private facility (PNB).

### Characteristics of eligible women for the CY program

Table 3 describes the overall characteristics of the study sample. The sociodemographic characteristics of women who delivered in an accredited CY facility and a non-private facility (PNB) did not significantly differ with the exception of proportions of women in the poorest (more in CY facilities) and richest (more in non-accredited private facilities) quintiles. Women who delivered in a government facility (GNP) were significantly poorer, less educated, higher parity, and belonged to tribes when compared to women who delivered in a CY facility. They also utilized antenatal care services less.

**Table 3:** Characteristics, pregnancy, and delivery details of the study sample (*n* = 901). Column % presented

	Accredited CY private facility	Non-accredited CY private facility	Government facility
No formal education	97 (34)	109 (39)	217 (65)**
Residence type (rural)	254 (89)	245 (88)	312 (93)
Caste			
Scheduled tribe (ST)	128 (45)	126 (46)	226 (67)**
Backward castes	153 (54)	136 (50)	102 (31)**
General	3 (1)	11 (4)	6 (2)
Household wealth			
1st quintile (poorest)	76 (27)	50 (18)^*^	83 (25)
2nd quintile	46 (16)	42 (15)	88 (26)**
3rd quintile	52 (18)	49 (18)	83 (25)**
4th quintile	59 (21)	61 (22)	45 (13)**
5th quintile (least poor)	53 (19)	77 (27)^*^	36 (11)**
Parity—primiparous	106 (37)	111 (40)	78 (23)**
Less than 3 antenatal check-ups	58 (21)	53 (20)	109 (36)**
Cesarean section (CS) delivery	22 (8)	55 (20)**	18 (5)
Direct obstetric complication (yes)	51 (18)	49 (18)	43 (13)
Length of stay for vaginal delivery (hour) median	18	12	25
Length of stay for CS (hour) median	55	76	136
CY program knowledge (yes)	211 (74)	74 (27)**	68 (20)**

Reference group: CY facility

*p < 0.05; **p < 0.000

The proportion of direct obstetric complications reported was similar for women across all three places of delivery; however, the CS proportion was significantly higher for women who delivered in a non-accredited private facility (PNB) (20 %, *n* = 55/279) compared to women who delivered in a CY (8 %, *n* = 22/286) and government facility (GNB) (5 %, *n =* 18/336).

#### CY program awareness

More than a third of all women (*n* = 353) had previous awareness of the program. While 74 % (*n* = 211) of women who delivered in a CY facility had prior knowledge of the program, only 27 % (*n* = 154) and 20 % (*n* = 68) of women who delivered in a non-CY private (PNB) and government facility (GNB) reported the same.

The accredited social health activist (ASHA), a village volunteer, was responsible for informing almost half (*n* = 172/353) of the women who knew about the CY program. Women also gained knowledge of the program from local community health workers (*n* = 97), relatives, and friends (*n* = 85) and other sources including the facility itself and the media (*n* = 52). Among the women who did not have prior knowledge (*n* = 541), half delivered in a government facility (GNB) and a third delivered in a non-accredited private facility (PNB)

### The proportion of CY beneficiaries

A third (*n* = 286/901) of the women in the study delivered in an accredited CY facility, but only half (*n* = 150/286) became program beneficiaries. As shown in Additional file 2, women who received the CY benefit did not significantly differ from non-beneficiary (CYNB) women who delivered in the same facility with the exception of education and prior knowledge of the program. Women who received the benefit were more educated and more likely to have had prior knowledge of the program. In a multivariable analysis (Additional file 2), formal education and knowledge about the program were significantly associated with receiving the CY benefit.

The main reasons cited by women who delivered in a CY facility but did not receive the benefit were lack of (i) proper documentation required by the provider to issue the benefit (*n* = 112) and (ii) awareness about the CY program (*n* = 46).

### Access to cesarean sections and blood transfusions

Among the 901 participants, a quarter (*n* = 233) delivered in a facility that did not routinely provide CS, 86 % (*n* = 201) of these were in government-operated facilities. Almost all of women who delivered in an accredited CY facility (*n* = 284) and 90 % of PNB (*n* = 249) had access to CS. Among women who delivered in a CY facility and non-accredited private facility, 70 % (*n* = 201) and 72 % (*n* = 201), respectively, had access to blood transfusion services as well.

### Out-of-pocket expenditures and degree of subsidy provided by the CY program

#### Out-of-pocket expenditures

**Fig. 2:**
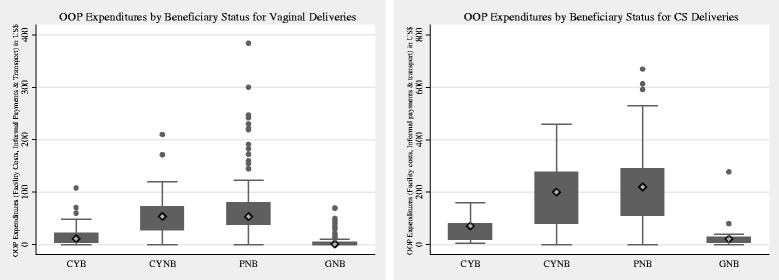
Boxplot for out-of-pocket expenditures by beneficiary status for vaginal and cesarean section deliveries (US$). *OOP* out-of-pocket, *CS* cesarean section, *CYB* CY beneficiary, *CYNB* delivered in an accredited CY facility, but did not receive the benefit, *PNB* private non-beneficiary, *GNB* government non-beneficiary

Figure 2 depicts the total OOP costs (health facility expenditure, informal payments, and transport) by type of delivery. Almost a quarter (*n* = 214) of the study sample women received a cashless delivery; the majority of these women delivered in a government facility (*n* = 178). Only 36 (24 %) of the 150 CYB received a completely cashless delivery. As described in Table 4, the median health facility expenditure for a vaginal/cesarean delivery among beneficiaries (CYB) who delivered in a CY-accredited facility was $5/$69 and $47/$199 for non-beneficiaries (CYNB). The facility OOP expenditures for CYB were significantly different than facility OOP expenditures for CYNB and PNB. The facility expenditures for women who delivered in non-accredited private facility (PNB) ($44/$208) did not significantly differ from non-beneficiaries (CYNB) who delivered in facilities where the CY program was operational. The median facility expenditure associated with government facility care was $0/$18.

**Table 4:** Median and interquartile range (IQR) for health facility expenditures associated with normal and cesarean deliveries, informal payments, and transportation costs in dollars

	CY beneficiary (CYB) median (IQR)	CY non-beneficiary (CYNB)^a^ median (IQR)	Private non-beneficiary (PNB) median (IQR)	Government non-beneficiary (GNB) median (IQR)
Health facility expenditure: vaginal delivery	5 (0–16)	47* (25–66)	44* (34–79)	0* (0–1)
Health facility expenditure: CS delivery	69 (20–78)	199* (81–274)	208* (102–284)	18 (6–28)
Informal payments (rewards)	0 (0–3)	2 (0–5)	0 (0–3)	0 (0–0)
Transportation	2 (0–6)	3 (0.6–10)	2 (0.4–6)	0 (0–1)

Conversion rate: US$1 = Rs 50. Reference group: CY beneficiary (CYB)

*CS* cesarean section

*p < 0.000

^a^Delivered in an accredited CY facility but did not receive the benefit

#### Degree of subsidy

The degree of subsidy provided by the program differed between vaginal and cesarean deliveries. The median subsidy for women who delivered vaginally was 85 % with an IQR of 74 to 100 %. Women who had a cesarean section received a median protection of 71 % and ranged from 66 to 80 %.

## Discussion

Previous literature on CY (i) has been small-scale studies performed during the initial rollout [21], (ii) has been on secondary data [24, 31], or (iii) did not identify CY beneficiaries [23]. Our study results show the uptake and level of subsidy provided by an innovative public-private partnership program to help remove financial barriers for poor/tribal women to deliver in a health facility. It contributes to the existing body of literature on the CY program and has not been reported previously with two main findings: (1) Uptake of the CY program was 16 % among eligible women and (2) the CY program subsidized a substantial portion of the cost for its beneficiaries. However, many eligible women were not able to avail the CY benefit despite delivering in a facility that participated in the program.

### Difficulty to reach the poorest populations

There is extensive literature establishing the link between poverty and maternal death. A study from Gujarat State found that poverty is the most important determinant influencing utilization of maternal health services, regardless of social caste or place of residence [32]. This inequality emphasizes the importance of developing innovative strategies that remove financial barriers and enable the most vulnerable women to receive proper access to delivery care. A recent synthesis of literature on demand-side financing programs argued that one of the most significant shortfalls of these programs is inadequate targeting, i.e., the difficulty to reach the poorest and underserved populations [33].

#### Low uptake in the Chiranjeevi Yojana program

There is a two-step process to receive the CY benefit: (i) Women must *choose* to deliver in a facility that participates in the program and (ii) then they need to *prove* their eligibility. We found only a third of the women chose to deliver in a facility that participated in the CY program, which implies that only a third of the study sample had the *opportunity* to become beneficiaries. Secondly, only half of those eligible women who delivered in a participating facility received subsidized services. Therefore, only a portion of our study sample successfully became beneficiaries despite a high awareness of the program in this group.

#### Steps to improve uptake

In light of the fact that most public facilities do not provide EmOC, uptake in the CY program needs to be improved so poor women have access to necessary care in the case of an obstetric emergency. While we do not know the explicit reason why women chose to deliver in a CY facility as we did not specifically enquire, a large proportion of women who delivered in a CY facility compared to non-accredited CY facilities had prior knowledge of the program. As highlighted in a recent review of maternal health voucher programs, community mobilization is one of the most important components of a successful program in terms of uptake and reaching the target population [19]. From our results, prior knowledge of the CY program was also a key determinant for receiving the benefit within a facility. Community level actors like the ASHA could be better utilized to improve awareness and knowledge of the program by targeting these vulnerable groups.

#### Barriers to receiving the CY benefit within a participating facility

As demonstrated in our study, delivering in a participating facility does not guarantee a woman will automatically receive the CY benefit. Lack of requisite documentation/proof of eligibility was reported as a barrier to receiving the benefit by many eligible women. In a qualitative study, Ganguly et al. found that some doctors who participated in the program felt that the women had little knowledge of the eligibility documentation needed to receive the benefit [27]. The role of and interaction with the community health worker becomes especially critical in the preparation of the documents necessary to establish the women’s eligibility status for the program and subsequently receive delivery services free of charge. The requirement to lower paperwork and documentation necessary for women to enter the program needs to be considered. Snarls around paperwork have been reported as precluding becoming a beneficiary.

### Is the Chiranjeevi Yojana program’s providing cashless deliveries?

While the program significantly subsidized delivery costs and reduced the financial burden for vulnerable women in our study through the CY program, only 36 of the 150 beneficiaries received a completely cashless delivery. Information asymmetry could be responsible for women not experiencing a cashless delivery. Similar to other health care settings in low-income countries, there is an extreme asymmetry between the health care provider and the patient [34].

Another probable explanation is the insecurity felt by some private health providers around receiving the reimbursement from the government. Some providers reported mitigating the risk of not receiving payment by imposing a cash deposit upon registration of the pregnant women for delivery. If the appropriate eligibility proof was supplied, the deposit was returned [27]. Constructive oversight in the form of better monitoring by the state can ensure cashless deliveries are facilitated under the program.

Poor uptake of the program could also be related to women sharing their experiences of paying for delivery services irrespective of the program’s intended objective. It is important for the program to ensure delivery services are free of cost at the point of care as this could be a deterrent for women to participate.

#### CY program reduces OOP expenditures for beneficiaries

A few studies have reported that childbirth expenditures, usually incurred in the private sector, are catastrophic for poor households [13, 35]. In our study, the CY program gave poor women the ability to choose where they delivered and receive EmOC if needed while avoiding debilitating amounts of debt. Even though a large majority of CY beneficiaries reported incurring some OOP expenditure, we still found a significant reduction in costs for those beneficiaries. Non-beneficiary women who delivered in a private facility paid 6.5 times more for a vaginal birth and three times more for a cesarean section than CY beneficiaries. This finding is consistent with what Bhat et al. previously reported (i.e., the CY program was effective in reducing OOP childbirth expenditures for its beneficiaries) [21]. However, Mohanan et al. found little or no association between the Chiranjeevi Yojana program and the reduction of OOP costs for deliveries [23]. The contradicting results may be explained by the fact they did not identify CY beneficiaries, the difference in study designs [36], and the timeframe when the OOP expenses were collected.

### Methodology considerations/limitations

This is the first study to estimate the proportion of CY beneficiaries among women who deliver in health facilities.

It has been reported in many Asian countries that families borrow money to pay for maternal-related costs thus being forced to forego essential items like food and education to repay the loans. These costs have a ripple effect on the family for years to come [37]. While this study has shown CY beneficiaries have reduced OOP expenditures compared to non-beneficiaries, it is not known if the reduction is large *enough*. Further research is needed to understand the magnitude of the reduction.

This study is facility based; therefore, our sample is restricted to women who reached a facility to deliver. While the proportion of home deliveries in Gujarat is low (10.7 %) [24], the majority of women who delivered at home would probably be eligible for the CY program.

Many studies highlight the limitations (e.g., recall bias and underreporting) associated with collecting health expenditure data [38–40]. Cost data was collected shortly after delivery and triangulated with other family members to minimize recall bias. A disaggregated cost collection design was used to improve accuracy and avoid underreporting of expenditures.

## Conclusions

CY program beneficiaries experienced a substantially subsidized childbirth compared to other women who delivered in non-CY-accredited private facilities. However, despite the government’s efforts at increasing access to delivery services for poor women in the private sector, uptake was low and very few women actually experienced a cashless delivery. While there is definitely a need to strengthen the provision of EmOC in the public sector, the CY program is a means by which the state can ensure its poor mothers have access to appropriate care at facilities that can provide EmOC. Measures need to be taken to improve uptake.

## Additional files

Additional file 1:
**Remuneration package for Chiranjeevi Yojana program at the time of the study.** (DOCX 14 kb) Additional file 2:
**Characteristics of CY Beneficiaries and CY Non-Beneficiaries who delivered in participating CY facilities and logistic multivariable regression for receiving the CY benefit (n=286).** Column % presented. (DOC 64 kb)
