# Supplementary material for: Statewide program to promote institutional delivery in Gujarat, India: who participates and the degree of financial subsidy provided by the Chiranjeevi Yojana program
Source: J Health Popul Nutr. 2016 Jan 27;35:2. doi: 10.1186/s41043-016-0039-z (PMC5026006; doi:10.1186/s41043-016-0039-z)
Supplement: Additional file 1: — Remuneration package for Chiranjeevi Yojana program at the time of the study. (DOCX 14 kb) [file 41043_2016_39_MOESM1_ESM.docx]

***Additional file 1:*** *Remuneration package for Chiranjeevi Yojana program at the time of the study.*

| **Procedure** | **Assumed cases per 100 deliveries** | **Cost in RS per procedure (US $)^1^** | **Total Rs (US $)** |
| --- | --- | --- | --- |
| **Normal delivery** | 85 | 1 800 (36) | 153 000 (3060) |
| **Complicated cases** |  |  |  |
| Eclampsia | 3 | 1 000 (20) | 6 000 (120) |
| Forceps/Vacuum/breech |  | 2 000 (40) |  |
| Episiotomy |  | 800 (16) |  |
| Septicemia | 2 | 4 000 (80) | 8 000 (160) |
| Blood transfusion | 3 | 1 500 (30) | 4 500 (90) |
| Cesarean section (7%) | 7 | 7 000 (140) | 49 000 (980) |
| **Pre-delivery Visit** | 100 | 100 (2) | 10 000 (200) |
| **Investigation** | 100 | 50 (1) | 5 000 (100) |
| **Sonography** | 30 | 150 (3) | 4 500 (90) |
| **NICU Support** | 100 | 100 (2) | 10 000 (200) |
| **Food** | 100 | 100 (2) | 10 000 (200) |
| **Transport** | 100 | 200 (4) | 20 000 (400) |
| *Total* |  |  | *280 000 (5600)* |

^1^ Conversion rate = US $1 = Rs 50, RS: rupees, NICU: neonatal intensive care unit
