# Supplementary material for: Statewide program to promote institutional delivery in Gujarat, India: who participates and the degree of financial subsidy provided by the Chiranjeevi Yojana program
Source: J Health Popul Nutr. 2016 Jan 27;35:2. doi: 10.1186/s41043-016-0039-z (PMC5026006; doi:10.1186/s41043-016-0039-z)
Supplement: Additional file 2: — Characteristics of CY Beneficiaries and CY Non-Beneficiaries who delivered in participating CY facilities and logistic multivariable regression for receiving the CY benefit (n=286). Column % presented. (DOC 64 kb) [file 41043_2016_39_MOESM2_ESM.doc]

***Additional file 2:*** Characteristics of CY Beneficiaries and CY Non-Beneficiaries who delivered in participating CY facilities and logistic multivariable regression for receiving the CY benefit (n=286). Column % presented.

|  |  | *CY Beneficiaries (*CYB) | *CY Non-Beneficiaries (*CYNB) | **Bivariable** | **Multivariable** |
| --- | --- | --- | --- | --- | --- |
|  | | n (%) | n (%) | OR 95% CI | OR 95% CI |
| **Districts*** | |  |  |  |  |
|  | Sabarkantha | 95 (60) | 63 (40) | 1.8 (1.0-3.4) | - |
|  | Dahod | 30 (41) | 43 (59) | 0.8 (0.4-1.7) | - |
|  | Surendranagar | 25 (45) | 30 (55) | *Reference* | |
| **No formal education** | | 38 (25) | 59 (43) | **0.4 (0.3-0.7)** | **0.5 (0.3-0.9)** |
| **Residence Type (Rural)** | | 135 (90) | 119 (88) | 0.8 (0.4-1.6) | - |
| **Caste** | |  |  |  |  |
|  | Scheduled tribe (ST) | 67 (45) | 61 (46) | 1.0 (0.6-1.6) | - |
|  | Backward castes | 81 (54) | 72 (54) | *Reference* | |
|  | General | 2 (1) | 1 (1) | 1.8 (0.2-20.5) | - |
| **Household Wealth** | |  |  |  |  |
|  | 1^st^ quintile (Poorest) | 35 (23) | 41 (30) | 0.8 (0.4-1.7) | - |
|  | 2^nd^ quintile | 29 (19) | 17 (13) | 1.6 (0.7-3.7) | - |
|  | 3^rd^ quintile | 28 (19) | 24 (18) | 1.1 (0.5-2.4) |  |
|  | 4^th^ quintile | 31 (21) | 28 (21) | 1.1 (0.5-2.2) |  |
|  | 5^th^ quintile (Least Poor) | 27 (18) | 26 (19) | *Reference* | |
| **Parity - Primi-Parous** | | 52 (35) | 54 (40) | 1.2 (0.8-2.0) | **-** |
| **Antenatal check-up (at least 3)** | | 119 (80) | 102 (78) | 1.2 (0.7-2.1) | - |
| **Cesarean Delivery** | | 9 (6) | 13 (10) | 0.6 (0.3-1.5) | **-** |
| **Direct Obstetric Complication (Yes)** | | 28 (19) | 23 (17) | 1.1 (0.6-2.1) | - |
| **CY Program knowledge (Yes)** | | 131 (87) | 80 (60) | **4.7 (2.6-8.4)** | **3.9 (2.1-7.3)** |
| **Program Documentation** | |  |  |  |  |
|  | Proper documentation not shown | 52 (35) | 61 (45) | *Reference* | |
|  | Proper documentation shown | 31 (21) | 14 (10) | **2.6 (1.3-5.4**) | 2.2 (1.0-4.8) |
|  | Scheduled tribe | 67 (44) | 61 (45) | 1.3 (0.8-2.1) | 1.2 (0.7-2.1) |

** row percentage presented*
